# The common ground of genomics and systems biology

**DOI:** 10.1186/1752-0509-8-S2-S1

**Published:** 2014-03-13

**Authors:** Ana Conesa, Ali Mortazavi

**Affiliations:** 1 Genomics of Gene Expression Lab, Centro de Investigaciones Príncipe Felipe, Valencia, Spain; 2 Department of Developmental and Cell Biology, University of California, Irvine, CA 92697, USA; 3 Center for Complex Biological Systems, University of California, Irvine, CA 92697, USA

## Abstract

The rise of systems biology is intertwined with that of genomics, yet their primordial relationship to one another is ill-defined. We discuss how the growth of genomics provided a critical boost to the popularity of systems biology. We describe the parts of genomics that share common areas of interest with systems biology today in the areas of gene expression, network inference, chromatin state analysis, pathway analysis, personalized medicine, and upcoming areas of synergy as genomics continues to expand its scope across all biomedical fields.

## 

The modern history of biological and medical sciences can be summarized in three words: ever-increasing specialization. As biologists have methodically surveyed the characteristics of living systems at the multiple scales of molecular, cellular, organismal, and ecological organization, seminal discoveries and technical advances have spawned entirely new fields of research that quickly develop their own themes, vocabularies, and research culture. Whether intended or not, this specialization usually involves reducing the scope of the problem to focus research and ease the burden of tracking too many variables and concepts. This reductionist approach is widespread to all sciences and no biologist or other biomedical scientist can claim deep knowledge of the state-of-the-art in more than a few of the hundreds subfields of biology. Running counter to this natural balkanization are the handful of organizing principles that span across all of biology such as natural selection and the central dogma of molecular biology. Why would anyone look for an alternative to reductionist biology after one hundred fifty years of unrelenting success?

Several subfields of biology have discovered independently that detailed studies of the structure and function of individual parts in detail did not give them a good understanding of the emergent properties of the interactions of many parts within a whole system. Neuroscience, physiology, and ecology all converged independently on the idea that it was as important to study and model the system of parts and their interactions as to fully analyze the individual parts alone. While system-centric studies in specific subfields of biology can be traced back to the 1950s and 1960s, it was not until the mid-1990s that systems biology developed into a major counter-movement that would grow to challenge the reductionist approach. A common definition of systems biology is the study of a given biological system by (a) the perturbation of a property of that system, (b) the measurement of resulting gene, protein, and pathway responses, (c) the integration of these data, and (d) the ultimate modeling of these data to describe the system as well as its response to perturbation [1]. We refer the reader to a review of the common ground of computational neuroscience with systems biology for a brief historical overview of the emergence of modern systems biology from pre-existing biological fields [2]. Fundamentally, the essence of systems biology is the study of interactions between parts of the system using experimental and computational methods.

The tremendous growth of interest in systems biology was driven by the simultaneous rise of genomics [1,2], which is the field dedicated to the large-scale analysis of the properties of genomes. As the international human genome sequencing project ramped up, the scientific community in concert with funding agencies devoted increasing efforts to the development of computational methods for genome assembly, annotation, and analysis. The concurrent development of microarrays as the first platform for large-scale gene expression measurements led to the birth of the new field of functional genomics, which quickly expanded to include other biomolecules, namely proteomics and metabolomics for the measurements of protein levels and metabolic intermediates respectively. A hallmark of this functional data is that they represent global measurements of thousands of molecular features where no one feature has an *a priori *higher importance than others. In this review we consider genomics in the broadest sense to include both structural and functional genome-wide measurements. In the context of transcriptomics, the mapping of transcripts onto exons on the genome is a structural measurement, whereas the expression levels of transcripts are functional measurements. The transformation of biology by genomics from a relatively data-poor into a data-intensive field has motivated the development of novel computational, machine-learning and other quantitative methods for genomic analysis that attracted a large number of engineers, physicists, and mathematicians into biology. As gene expression and other functional data have accumulated through ever-larger scale projects such as ENCODE [3], significant efforts have been invested in integratively analyzing data to build gene regulatory networks [4,5]. Most models built from high-throughput genomics data tend to be correlative with relatively limited predictive power. This version of systems biology that emphasizes parts (a)-(c) of the definition above is barely recognizable to other biologists who associate systems biology with a more mathematical modeling driven approach that attempts to explain biological phenomena of a system with a limited number of parts using differential equations, which emphasizes part (d) of the definition. This difference of opinion leads to passionate discussions of whether genomic analyses qualify as systems biology or do not. We believe that, in the broadest sense, many parts of genomics do fall within the purview of systems biology. We do not attempt to give here an exhaustive review of genomics or systems biology because of the vast literature of each field. Instead, this review delineates explicit areas of overlap between genomics and systems biology related to transcriptomics, metabolomics, and gene regulatory network inference as well as outlines some of the genomic challenges that will likely drive the field forward.

### Defining the overlap between genomics and systems biology

Why not consider all of genomics or at least all of bioinformatics to be part of systems biology? After all, most genomic experiments generate thousands to billions of data points that require quantitative, bioinformatic methods for analysis. However, there are bioinformatic tasks that provide little direct insight into a system without further analysis. For example, the mapping of reads and the assembly of genomes are two critical, foundational activities of genomics that pose some of the greatest algorithmic challenges and are very active areas of research. Yet the resulting assembly or the location of reads onto a genome are not informative on their own about the system, but require further analysis with additional tools. We can use the operational definition of systems biology as the study of interactions between parts of the system to identify areas of genomics that are clearly systems-centric and others that are more dependent on the goals of the experiment. For example, while sequencing a transcriptome solely for discovery of novel transcripts would not fall within the realm of systems biology, the analysis of the change of gene expression in existing and newly-discovered transcripts during a developmental time course or after a perturbation such as an siRNA certainly would qualify. Similarly, the identification of SNPs in an individual genome would not qualify as systems biology, but the quantification of their effect on the expression of associated genes and the identification of gene expression Quantitative Trait Loci (eQTL) [6,7] definitely does. The sequencing of cancer genomes to identify the mutations driving the cancer represents a third such systems approach of how changes in one part of the system affects the behavior of the whole system.

We can broadly describe the many subfields of genomics as falling under three over-arching categories based on their relationship with the genome under study on a continuum of "pure" to "applied" genomics: global, general, and specific (Figure 1). Sub-fields of genomics that focus on a global view are those that provide a single answer that is essentially identical for all individuals from that species. This would include the reference genome assembly and annotation as well as comparative genomics (Figure 1). While algorithmically challenging, these analyses do not shed light on the behavior of the system per-se, but represent more a catalog of the parts, which can later serve as a starting point for systems-level analyses. The second category encompasses fields of genomics that are interested in analyzing specific aspects of the genome "in action" such as transcriptome discovery in a specific cell-type, or tissue, or the analysis of the encoding of the transcriptional logic of gene regulatory networks underlying development. These problems can be generally reframed within a systems framework to get insights into their function and behavior. For example, the study of the dynamics of gene regulatory networks represents one of the primary problems in the field of transcriptional regulation that is also a classical example of systems biology [1]. Finally, there are a multitude of genomic experiments that are specific to the factor, or cell type, or individual under study (Figure 1). This includes examples such as transcription factor interactomes measured using ChIP-seq [8], transcriptome quantitation using RNA-seq [9], and genome variation analysis in individual genomes [6,7]. A characteristic of these problems is that they typically require associating called peaks, expression levels, or variants to specific genes and inferring functional enrichment in pathways using tools such as pathway analysis [10,11] and Gene Ontology [12], which fall under our definition of systems biology. In our post-Sanger-sequencing world, another characteristic of genomic problems suited for systems biology is that their starting data typically comes in the form of millions of data points such as short reads that enable statistically analyzable counting assays. While global problems such as genome assembly will always benefit from ever longer reads, counting assays benefit primarily from additional reads rather than longer read length (Figure 1), as discussed in a separate review [13]. We now turn to a more detailed analysis of individual genomic fields and their relationship to systems biology (Figure 2).

**Figure 1:**
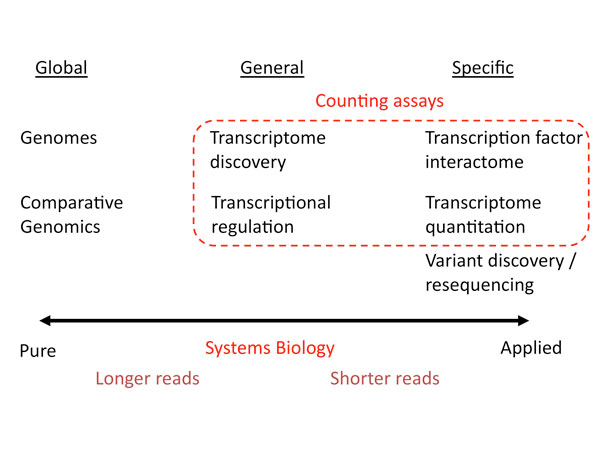
**The continuum of genomics problems**. Representative areas of genomics are arranged along a continuum of pure to more applied genomic research problems that can be grouped into three overall categories of global, general, and specific problems with respect to the genome under study. In this schema, genomic problems that can benefit from a systems biology approach generally fall under the general and specific columns and typically rely on counting assays that leverage the large number of reads or datapoints generated by modern high-throughput platforms.

**Figure 2:**
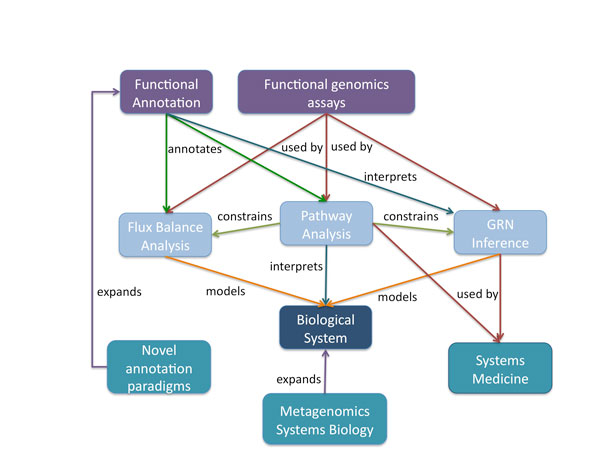
**Relationships between systems biology and genomics**. Functional genomics assays like gene expression profiling, metabolomics and proteomics are used as input data by different systems-level analysis approaches such as Gene Regulatory Network (GRN) inference, Pathway Analysis (PA) and Flux Balance Analysis (FBA). Functional annotation, a core activity of genomics, is a prerequisite in PA and FBA, and helps in the interpretation of GRNs. GRN and FBA generate models of the biological system based on genomics data and can also use pathway databases as *a priori *information to help building models of the system. Alternatively, PA can be directly employed as an interpretative tool of the system. Systems Medicine relies on GRNs and pathways to develop personalized genomic diagnosis tools. Metagenomics expands the system under study to a supraorganismal level, whereas novel systems-level annotation paradigms such as transcript annotation expand the scope of functional annotations.

### Gene expression

Whereas the elements that make up the genetic definition of living organisms are encoded into the genome, it is the ensemble of expressed genes that are the actual manifestation of the biological system. Regardless of how gene expression is regulated, expressed transcripts are prerequisite, primary components of cell physiology. The development of genome-wide gene expression profiling technologies, using microarrays first and sequencing since, has brought the analysis of gene expression into the realm of systems biology. There exist a variety of techniques such as arrays, SAGE, CAGE, and RNA-seq that allow for different combinations of quantitative (transcript expression levels and differential expression) and/or discovery (splicing events and transcript intron/exon organization) analyses of transcriptomes. While the measurement of expression genome-wide is only the first step in deriving system-level knowledge, it presents analytical challenges to this day. The main reason is that genomic experimental techniques measure individual parts of the system in parallel but cannot directly measure the system structure, which needs to be inferred. This inference is complicated by the high variable to observation ratio of genomics, which causes the intrinsic and heavily underdetermined nature of the genomics/systems biology marriage. Experimental designs involving time courses and or perturbation can provide significantly more, but rarely enough, information on the underlying system structure. Computational biologists address this high underdetermination problem using strategies such as variable selection [14-17], model constraints by additional data [18] or exhaustive search of the results space to reach local optimal solutions [19-21]. However, there are still methodological and conceptual limitations that must be overcome to bridge the gap between simple gene expression analysis and the inference of molecular systems. For example, most of the popular differential gene expression methods that are used for variable selection provide single gene-based assessments of differential expression [22-25]. While these methods incorporate parameterizations to account for the high dimensional nature of genomics data, such as pooled variance estimates [22] or multiple testing correction [26], they completely ignore the interactions of genes as parts of large-scale biological pathways and systems [27]. A first step in this direction is the application of multivariate methodologies to transcriptome analysis that exploits the covariance structure of the expression data matrix to infer patterns of gene expression and select genes for their relevance in those patterns [28,29]. The underlying hypothesis here is that covariance is a proxy of co-expression and that relevant processes (and genes) of the system can be identified for their co-expressing characteristic. Approaches that come closer to a systems driven analysis of differential expression have used gene network data to guide the multivariate analysis under the assumption that genes for which an interaction exist are correlated in their differential expression states [30,31] or have taken an Empirical Bayes approach by modeling networks as a Markov random field (MRF) to identify genes and sub-networks that are related to diseases [32,33].

### Network inference as the common core of genomics and systems biology

When considering the interplay between systems biology and genomics, two major paradigms stand out: one is the use of gene expression measurements to obtain the structure of the system and infer Gene Regulatory Networks (GRNs), while the other is the leveraging of system properties to interpret observed gene expression patterns using pathway enrichment methods (Figure 2).

The systems biology of gene expression is frequently understood as a problem of gene regulatory network inference, where gene networks capture how the expression profile of individual genes interacts with each other [1]. The encoding and dynamics of transcriptional regulation have fascinated scientists ever since the seminal work of Jacob and Monod on the *lac *operon in *E. coli *[34]. The last fifty years have clearly shown that the transcriptional regulation is encoded in GRNs that robustly control spatiotemporal expression of genes to enable proper development and function from the simplest bacteria to the most complex animals and plants [35]. Popular systems-based approaches such as Weighted Gene Correlation Network Analysis (WGCNA) have been applied successfully to a variety of biological settings to identify modules of tightly co-expressed genes in cases such as single-cell human and mouse RNA-seq time-courses of early pre-implantation embryonic development [36]. Recent efforts to build genome-wide GRNs from transcription factor ChIP-seq [4] and DNase-hypersensitivity data [5] from multiple cell types have heavily focused on the regulation of the regulators such as transcription factors and other signaling-related genes. These regulatory proteins form the core of GRNs with complex, intertwined feedback loops between regulators at the transcriptional and often post-transcriptional level. Systems-based techniques play a key role in the analysis of GRNs as it is nearly impossible to understand the behavior of a moderately complex GRN that incorporates feedback loops without modeling. As it becomes increasingly practical to map the regulatory linkages in GRNs from large-scale functional sequencing data, the challenge of modeling and predicting the dynamics of GRNs becomes ever more pressing.

The rapid increase in the amount of multiple, complementary chromatin-related data in the same sample such as ChIP-seq of different histone modifications and transcription factors as well as DNase-seq has led to the development of a new set of tools to analyze the data integratively in order to learn more about the global organization of the genome. Two very different approaches have been used to analyze such data, which is typically preprocessed into genomic segments with boundaries derived from the data signal. The first approach uses the chromatin data on the segments for training of Hidden Markov Models [37] or Dynamic Bayesian Networks [38] to learn the smallest number of states that can recapitulate the major processes of transcriptional regulation and effectively annotate the genome *de novo*. The hidden states from these models are learned from the data and their chromatin signatures are interpreted post-training to associate particular states with promoters, enhancers, transcribed regions, or repressed regions. While there is no *a priori *defined number of states that we can safely expect in the genome, smaller numbers of states (preferably less than 20) are often preferred for the sake of interpretability. However, the combinatorial nature of gene regulation points to another extreme, where we are interested in identifying relatively small cohorts of genomic regions that show similar coordinated changes of chromatin marks and transcription factor binding across many data sets and multiple cell types. In such cases, we would like to interrogate the genome with a much larger number of potential micro-states and then apply some form of dimension reduction to identify related micro-states that form larger coherent groups of "meta-states". A Self-Organizing Map (SOM) is another unsupervised machine learning clustering technique that has been used in two recent publications to analyze a large number of ChIP-seq (and DNase-seq) datasets using maps with potentially at least a thousand such micro-states [39,40]. The maps consist of thousands of units (or "neurons") that are arranged in a two dimensional grid. In order to avoid boundary effects, the maps are often laid on the surface of a toroid that can be unwrapped for visualization. Each unit of the map has an associated vector that is originally initialized randomly. The map is trained using the vectorized signal from the datasets (either binarized [40] or using RPKM signal density [39]) for each segment until the map converges. Every segment is then assigned to the best matching unit on the map. The resulting map is mined for relationships between training dataset enrichments in specific units and can be interpreted further by laying additional data on the map not used during the training. These maps typically reveal very distinct colocalization patterns between particular datasets in specific cell-types. While the results from the hidden-state-based or SOM-based approaches are global, they can both be mined to identify the actual, underlying regulatory elements encoding the GRNs and will presumably be used for further automated attempts to derive networks from functional sequencing data.

However the concept of molecular networks extends beyond gene regulatory networks. In fact, much of the early research in systems biology focused on flux balance analysis (FBA), which is a genome-wide analysis of metabolic regulation [41,42]. FBA relies on simple stoichiometry rather than difficult to measure enzyme kinetics to analyze the behavior of metabolomics networks. FBA employs a linear programming (LP) strategy to generate a flux distribution that is optimized toward a particular 'objective', normally maximal cell growth, subject to a set of underlying physicochemical and thermodynamic constraints fitting experimental data on changes at nutritional and metabolic levels [43]. FBA can be integratively analyzed with genome-wide data by incorporating gene expression measurements into metabolic modeling (Figure 2). This combination enables the characterization of the regulatory modalities governing metabolism and for the identification of metabolic hubs [44-46]. For example, an analysis of yeast strains grown in different nutritional conditions combined Z-scores of metabolic fluxes obtained by either metabolic or gene expression measurements to classify the regulation level of metabolic circuits as transcriptionally, post-transcriptionally, or metabolically controlled [46]. In another study, FBA and gene expression were combined to predict the impact of 75 different drugs, drug combinations, and nutrient conditions on mycolic acid biosynthesis capacity in *M. tuberculosis*, using a public compendium of over 400 expression arrays [44]. The authors showed that e-Flux (expression and flux) analysis can be used to correctly predict the modulators of metabolite biosynthesis and the metabolic state under specific nutritional or treatment conditions.

The combination of metabolic modeling and gene expression analysis is not only relevant for drug target discovery, but is also of major importance for targeted metabolic engineering and synthesis of economically relevant compounds, energy production or waste treatment [47,48]. This economic potential, together with the development of cost-effective sequencing technologies, has boosted the sequencing of the genomes of novel microorganisms for biotechnology applications. A key element of the success of these approaches is the availability of efficient genome and annotation algorithms that characterize the metabolic potential of the newly sequenced genomes (Figure 2). Reference functional databases such as KEGG [49] and AraCyc [50] are frequently used as the backbone for metabolic reconstruction, which needs to be further complemented by algorithms that build the genome-wide metabolic network, fill in reaction gaps and validate predictions [51]. In this sense genome (functional) annotation, a core activity of genomics, is a necessary prerequisite for the computation-based reconstruction of the metabolome of novel species and hence serves as a substrate for systems-level analyses of genomic data (Figure 2).

### Pathway analysis as the interpretative tool of systems biology

The inference of gene and molecular networks is focused on mapping the mechanistic and structural properties of the system. Genome-wide gene network analyses typically produce large networks that involve hundreds of gene interactions. Such networks might have interesting topological properties that are biologically meaningful, but are normally difficult to interpret in terms of cellular functionality. Functional enrichment analysis methods (also referred to as pathway or gene set enrichment) are methodologies that allow us to analyze gene expression data for the biological meaning of particular expression patterns in order to gain additional insight into the actual biology of the system [52-54] (Figure 2). These functional assessment methodologies rely on the premise that the expressed components of cellular systems are likely to be functionally coordinated and that genes belonging to the same functional unit should show similar expression profiles. The first functional enrichment analysis methods identified pathways that were overrepresented within a list of differentially expressed genes [52] and were rapidly followed by the gene set enrichment approach [53] where a ranked, rather than a selected, list of genes was used to find associations between phenotypes and cellular functions. There are now a multitude of implementations based on this concept that introduce additional functional data such as protein interaction data [55,56], gene regulatory networks [57], pathway topology information [58], metabolic changes [59,60] or expression kinetics [61]. These methods have been applied not only to understand gene expression changes but also in Genome-Wide Association Studies (GWAS) [62], comparative genomics [63] and gene prioritization [64].

### Personalized medicine and other upcoming challenges

The rapid availability of ubiquitous sequencing holds great promise for medicine to the extent that genomics empowers the analysis of patient genomes to guide personalized treatment. While we can now sequence an individual's genome and transcriptomes, it remains extremely difficult to use that data to inform treatment. We currently lack the capability to evaluate the impact of most sequence variants found and what their functional consequences are. A decade of GWAS studies have revealed a multitude of common variants associated with various traits and diseases, each of which seems to contribute to or at least to increase the probability of a phenotype by a small amount [65]. Yet most of these variants are in non-coding regions and we are often not even certain of the association of the variant with an actual gene. Parallel efforts by projects such as ENCODE [3] to annotate the functional parts of the genome have highlighted the functional complexities of the genome beyond coding sequences. A recent study found that 76% of non-coding GWAS SNPs associated with various phenotypes or diseases are found within or in perfect linkage disequilibrium with DNase hypersensitive sites called within ENCODE and the NIH Epigenomic Roadmap Project, which suggests that they are associated with functional regulatory elements [66]. While this is highly encouraging, we are still unable to assess the contribution of these changes in functional elements to what are often complex phenotypes that arise from these combinatorial interactions between multiple variants occurring jointly at genes, let alone their interactions with the environment. Adopting methods from systems biology to marshall the data into tractable, predictive models can shed light on the contributions of these individual variants to the phenotypes under study. For example, an interesting application of system biology to personalized medicine was the application of a flux balance analysis (mostly used in prokaryote metabolic reconstruction) for modeling the metabolism of a single Hereditary Hemorrhagic Telangiectasia patient to identify altered metabolic fluxes and to devise a personalized treatment that eventually improved patient condition [67] (Figure 2).

A particularly compelling set of use cases for the application of systems biology to understand the genomics of disease can be found in cancer. Efforts to characterize the most prevalent mutations of various cancers by The Cancer Genome Atlas (TCGA) Research Network have revealed recurrent mutations in specific pathways. The recent TCGA Lung Squamous Cell Carcinoma (LSQCC) study [68] represents a particularly nice example of what can be accomplished by combining genome and transcriptome sequencing with systems-level pathway analysis. The sequencing of 178 patient samples found that in addition to universal mutations to TP53, each cancer carried higher-order combinations of multiple reoccurring mutations. LSQCC was divided into four subtypes based on a combination of expression, copy number variation, and methylation. Most promisingly, the authors found recurring mutations within targetable oncogenic pathways such as PI(3) kinase, RAS, and Receptor Tyrosine Kinases. While it is rare that cataloguing mutations in cancer alone will reveal both mechanisms of disease progression and potential drugable targets, we are left with the greater challenge of understanding how some cancers can relapse after treatment. One possible solution is the use of network concepts to identify groups of genes that when perturbed give the same phenotype and hence form a disease module [69]. If systems biology can rise to the challenge of predicting the mutations that are most likely to allow a cancer to relapse, we may be able to design multi-drug treatments that will prevent cancers from evading conventional drug treatments. More generally, systems biology holds the promise of helping to decrease the time and costs of developing new drugs and also helping to provide more targeted and safe candidate drugs by leveraging pathway analysis. At the same time, the new field of pharmacogenomics seeks to understand the interactions between drugs and individuals' genotype. For example about 14% of the population carry the *2 allele of the cytochrome P450 CYP2C19 that prevents the proper processing of the anti-clotting drug clopidogrel (Plavix) and thus renders the drug ineffective [70]. Just as in the case of cancer genomics, we need to use systems biology approaches if we are to capitalize on the patient's genome to identify how variants interact with drugs and predict what the ultimate effectiveness of these drugs might be in a specific patient rather than averaged over the whole population.

Another area of great promise for mutual reinforcement between systems biology and genomics is in the study of the composition and interactions of bacterial communities with their environment (Figure 2). A variety of sequencing projects have revealed that large numbers of uncharacterized microbial species cooperatively interact in the environment in every imaginable ecological niche. This includes the microbial communities that are associated with specific human body niches and are characteristic of several human conditions such as Inflammatory Bowel Disease (IBD) and obesity [71]. While we are accumulating large metagenomic datasets and cataloguing bacterial genomes that make up the different parts of the human microbiome in normal and diseased individuals, it is still very difficult to connect the presence or change in frequency of specific bacterial species with the associated phenotypes. An early example of metagenomic systems biology beyond simple comparative studies treats the entire metagenome as a single system and analyzes the changes in metabolic networks inferred from topological models of healthy and diseased metagenomes in IBD and obesity [72]. The ultimate challenge will be to model the interactions of the microbiome community with the host.

Functional annotation is, as pointed earlier, a fundamental substrate of systems biology. Functional annotation provides *a priori *knowledge, interaction constraints and an interpretative framework for systems biology (Figure 2). More effective methods for functional annotation are necessary to leverage further genomic data for system-level analyses. For example, nearly half the genes of higher eukaryotes are proteins of unknown function or non-coding genes that await functional characterization. High throughput screening methods for protein-coding genes [73,74] or computational predictive approaches in the case of non-coding transcripts [75] might help to speed up functional characterization but are still at their infancy and far from being generally applicable. Moreover, the systems-oriented analysis of gene expression still has much to evolve both methodologically and conceptually. For example, pathway methods rely on existing annotation data that points to which genes are involved with specific cellular roles, but most annotation databases are static and do not incorporate tissue or development specific information. Moreover, the assignment of genes to functions is still a largely unfinished task and the boundaries of pathway definitions are arbitrary: one database might include a set of genes within a specific signaling pathway while another would split this into two separate pathways. The best way to reconcile different pathway views and to capture the plasticity of signaling and metabolomic pathways is still an open question in genomics research. Additionally, functional enrichment methods typically consider all genes in the gene set as equally contributing to the functional capacity of the set, thereby ignoring the stronger regulatory role of some pathway components and hence their differential impact on the pathway functionality. Relevant pathway genes could be identified by their network properties as it is done in systems medicine [68] or by being highly regulated in Pathway Network Analysis [76]. This strategy is predicated on the concept of driving genes that account for most of the variability in the coordinated expression of the pathway and are major contributors to changes in pathway activity [76]. However, there is still a need for accurate systematic approaches to dissect the differential relevance of genes within pathways. As gene expression analysis continues the transition to high-throughput sequencing, transcripts rather than genes become the fundamental feature measured and will require the update of functional profiling methods to support transcript-level functional analyses of enrichment. In particular, the analysis of the functional consequences of alternative-splicing within a systems framework, such as the analysis of the exonic targets of the neuronal splicing factor NOVA1 [77], remains a relatively unexplored area that seems destined for advances with more accurate transcript reconstruction methods from RNA-seq data. Interestingly, as system biologists and bioinformaticians build widely used tools for pathway analysis and differential gene expression, their end users do not necessarily consider themselves to be doing systems biology explicitly, even though they publish system-level analyses in their publications. For example, the pathway analysis tool PARADIGM is designed to find pathway-level changes in cancers using graphical models that clearly fall within the scope of systems biology [78], yet its users in TCGA (such as in [68]) do not claim to take explicit systems approaches. We take this to be a sign of the success of system-level analyses.

Although genomics and systems biology have started to reshape a multitude of areas in biology, new subfields evolve with ever more specialization. Ironically, we suspect that systems biology and genomics are in fact contributing to a new era of specialization by creating entire new subfields such as developmental systems biology or pharmacogenomics. However, as genomics continues to expand and to mature by addressing nearly every imaginable biological question, it is increasingly clear that the primary analysis of the resulting data alone is no longer sufficient for extracting new biological insights. Instead, we need to leverage the ideas and techniques of systems biology to understand the behavior of the system and its multitude of parts. Similarly, the challenges that genomics is now tackling by integratively analyzing ever higher-dimensional, multi-species systems will likely require the development of more sophisticated hierarchical models by the systems biology community to enable meaningful joint comparative analyses. Last but not least, genomics will need to leverage systems biology by building predictive models from personal genome data to produce actionable results for patient care that delivers on the promise of precision medicine. Thus there is much more work to be done jointly by genome and systems biologists.

## Competing interests

The authors declare that they have no competing interests.

## Authors' contributions

Both authors contributed equally to the manuscript.

## Declarations

The publication costs for this article were funded by MINECO grant BIO2012-40244 and FP7 STATegra number 306000.

This article has been published as part of *BMC Systems Biology *Volume 8 Supplement 2, 2014: Selected articles from the High-Throughput Omics and Data Integration Workshop. The full contents of the supplement are available online at http://www.biomedcentral.com/bmcsystbiol/supplements/8/S2.
